# Development and Biomechanical Evaluation of a Modular Knee Prosthesis: From Conceptual V1 Design to an Improved V3 Model

**DOI:** 10.3390/bioengineering13020201

**Published:** 2026-02-11

**Authors:** Samal Abdreshova, Sayat Akhmejanov, Kassymbek Ozhikenov, Nursultan Zhetenbayev, Yerkebulan Nurgizat, Dauren Bizhanov, Aidos Sultan, Abu-Alim Ayazbay, Meruert Zharmagambetova, Gani Sergazin

**Affiliations:** 1 Department Aerospace and Electronic Engineering, Almaty University of Power Engineering and Telecommunications, Almaty 050013, Kazakhstan; s.abdreshova@aues.kz (S.A.); y.nurgizat@aues.kz (Y.N.); d.bizhanov@aues.kz (D.B.); a.ayazbay@aues.kz (A.-A.A.); 2 Department of Robotics and Technical Tools of Automation, Satbayev University, Almaty 050013, Kazakhstan; akhmejanov.s@stud.satbayev.university (S.A.); k.ozhikenov@satbayev.university (K.O.); a.sultan@aues.kz (A.S.); 3 Department of Science and Innovations, Mukhametzhan Tynyshbayev ALT University, Almaty 050013, Kazakhstan; m.zharmagambetova@alt.edu.kz (M.Z.); g.balbayev@alt.edu.kz (G.S.)

**Keywords:** knee prosthesis, modular prosthetic system, CAD modelling, kinematic analysis, motion simulation, biomechanical design, lower-limb prosthetics

## Abstract

This study investigates the functional capabilities and accessibility limitations of current knee prostheses while developing and evaluating a three-stage prosthetic system (V1–V3). The primary objective is to design a cost-effective knee prosthesis featuring anatomically compatible motion, high kinematic accuracy, and a modular architecture. The methodology integrates a technical review of commercial prostheses, CAD modeling in SolidWorks, kinematic evaluation through Motion Simulation, and experimental testing of the V2 prototype. The results demonstrate the structural limitations of the initial V1 design, the complete assembly and improved functional performance of the V2 prototype, and the advanced mechanical behavior achieved in the final V3 concept. The V3 model provides an extended range of motion, reduced mass and lowered center of gravity, smoother dynamic response, and compatibility with a fully modular foot–ankle–knee configuration. Overall, the findings indicate that the V3 design represents a promising engineering solution that brings the system closer to clinical applicability and establishes a foundation for the development of a fully modular lower-limb prosthetic platform.

## 1. Introduction

Knee joint amputation remains a major challenge in rehabilitation engineering due to its profound impact on human locomotion, stability, and energy efficiency during walking. The knee joint plays a critical role in load transfer, shock absorption, and phase-dependent motion control throughout the gait cycle. Any disruption at this level directly alters gait symmetry, joint coordination, and overall mobility, often leading to secondary musculoskeletal complications and reduced quality of life. Consequently, restoring knee joint function through prosthetic systems is not merely a mechanical problem, but a biomechanical and control-oriented challenge that lies at the intersection of human locomotion theory and engineering design [1,2,3].

From a biomechanical perspective, the knee joint exhibits highly nonlinear behavior, with variable stiffness, damping, and torque requirements across stance and swing phases. Accurate reproduction of these characteristics is essential for achieving stable gait and minimizing metabolic cost. Numerous studies have demonstrated that inadequate knee control leads to gait deviations, increased energy expenditure, and elevated fall risk, particularly during transitional tasks such as stair ascent, descent, and uneven terrain walking [4,5,6,7]. These findings highlight a fundamental scientific question: how can prosthetic knee mechanisms approximate the adaptive biomechanical behavior of the human knee while remaining structurally and economically feasible?

Existing prosthetic knee technologies attempt to address this challenge through different design philosophies. Passive mechanical knees rely on spring–damper elements and geometric constraints to ensure stance stability, offering robustness and affordability but limited adaptability [8,9]. Microprocessor-controlled knees (MPKs) introduce sensor-based control strategies that modulate damping in real time, significantly improving safety and gait smoothness [10,11,12]. More advanced powered and hybrid systems actively generate joint torque using electric or pneumatic actuators, enabling closer replication of physiological knee function [13,14,15]. However, despite their biomechanical advantages, such systems are often associated with increased complexity, weight, energy consumption, and cost, which restricts their accessibility and long-term reliability [16,17,18].

Recent research has therefore shifted toward adaptive control strategies, intelligent sensing, and modular architectures. Studies employing impedance control, adaptive frequency oscillators, and iterative learning methods have demonstrated improved gait synchronization and energy efficiency [19,20,21,22]. Parallel efforts in neural-network-based and EMG-driven control aim to infer user intent more accurately, further enhancing natural interaction between the user and the prosthesis [23,24,25]. At the same time, advances in materials, joint kinematics, and durability analysis continue to refine the mechanical foundations of prosthetic knees [26,27,28,29]. While these contributions represent significant progress, the literature reveals a persistent gap between biomechanical fidelity, system modularity, and economic viability.

In particular, many high-performance prosthetic knees integrate tightly coupled mechanical and control components, making them difficult to modify, scale, or adapt to different users and clinical contexts. This lack of modularity limits iterative development and hinders the systematic evaluation of design trade-offs between kinematics, control complexity, and manufacturability. As highlighted in recent reviews, there remains a need for prosthetic knee systems that combine biomechanically informed joint behavior with simplified mechanical architecture and clear evolutionary development pathways [30].

In response to this challenge, the present study addresses the following research objective: to establish an evolutionary engineering framework for the development of a modular knee prosthesis that balances biomechanical accuracy, structural simplicity, and future extensibility. Rather than presenting a single finalized device, this work introduces a three-stage evolutionary design pathway:

− V1 conceptual CAD-based structural design and preliminary validation;
− V2 physical prototyping and experimental assessment;
− V3 refined joint mechanism with expanded kinematics and a transition toward a fully modular architecture.

By systematically analyzing the progression from V1 to V3, this study highlights how biomechanical requirements, kinematic constraints, and engineering trade-offs shape prosthetic knee design. The results contribute to a clearer theoretical and practical understanding of how modular, biomechanically compatible knee prostheses can be developed as part of an integrated foot–ankle–knee system.

### 1.1. Biomechanical Fundamentals of the Knee Joint and Human Gait

**Figure 1:**
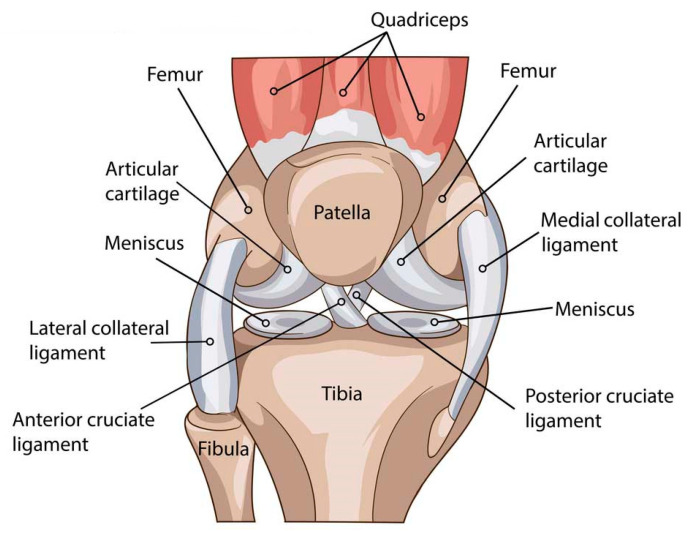
Anatomical structure of the human knee joint.

The knee joint (articulatio genus) is one of the largest and most complex structures of the human musculoskeletal system. It is formed by the medial and lateral condyles of the femur, the tibial plateau, and the patella. The primary functions of the knee include supporting body weight during standing, walking, and running; ensuring safe and efficient load transmission; and enabling a wide range of flexion–extension movements. The joint performs two major motions, flexion and extension, while also permitting minor internal and external rotational movements due to its anatomical configuration. Knee stability is maintained by the cruciate ligaments (ACL, PCL) and collateral ligaments (MCL, LCL), which define the joint’s kinematic limits and serve as key passive stabilizers under load (Figure 1) [31,32,33,34].

From a kinematic perspective, the knee is often described as a hinge joint (ginglymus), although its motion is governed by a complex rolling–sliding mechanism. During flexion, the femoral condyles roll and simultaneously glide over the tibial plateau, resulting in a continuously shifting instantaneous center of rotation. This mechanism enables efficient shock absorption, energy conservation, and stable load transfer during locomotion.

In addition to flexion/extension, physiological knee motion involves coupled axial rotations and frontal-plane deviations. Femorotibial torsion occurs primarily during the stance phase as a result of internal/external tibial rotation relative to the femur, contributing to joint stability and load redistribution. Furthermore, varus thrust, a transient lateral deviation of the knee observed during early stance, is a well-documented biomechanical phenomenon associated with asymmetric load transmission across the medial and lateral compartments of the knee. These coupled kinematic behaviors play a critical role in defining realistic boundary conditions for knee joint modeling and are therefore considered implicitly in the geometric and kinematic assumptions adopted in this study.

**Figure 2:**
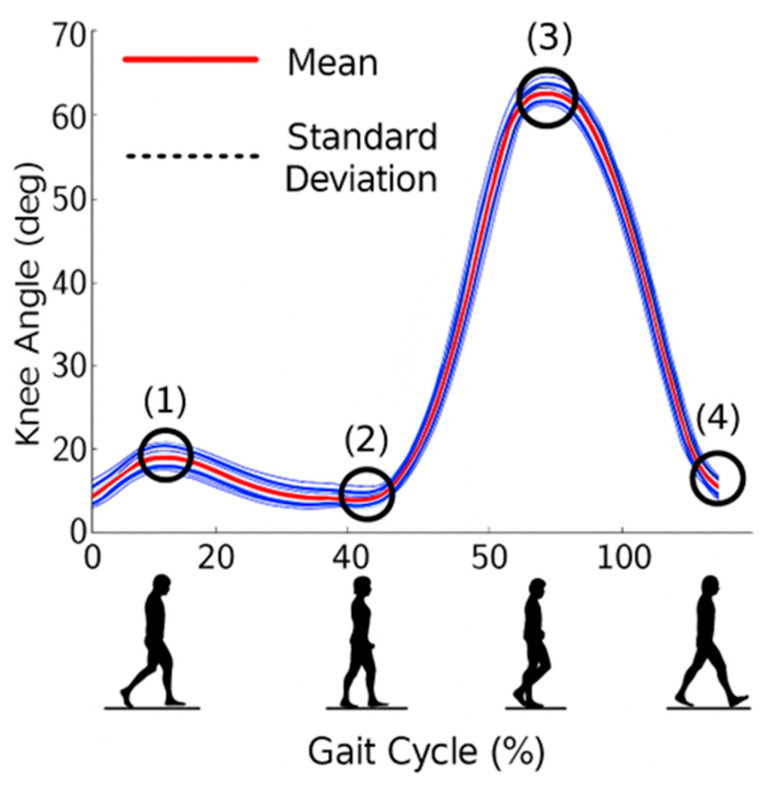
Knee flexion–extension angle profile over a complete gait cycle. The red curve represents the mean knee flexion–extension angle, while the blue curves indicate the variability of the measured data across repeated gait cycles. The shaded blue region reflects the dispersion around the mean profile. The numbered markers (1–4) denote characteristic gait events: (1) initial contact with low knee flexion; (2) loading response with slight flexion for shock absorption; (3) mid-swing phase corresponding to peak knee flexion for foot clearance; and (4) terminal swing phase, during which the knee extends in preparation for the next initial contact.

Human gait consists of two major phases: the stance phase (approximately 60% of the gait cycle) and the swing phase (approximately 40%). The stance phase begins with initial contact and ends when the contralateral foot strikes the ground and is subdivided into initial contact, loading response, mid-stance, terminal stance, and pre-swing. The swing phase encompasses limb advancement and preparation for the subsequent step (Figure 2) [35]. In Figure 2, the red curve represents the mean knee flexion–extension angle over the gait cycle, while the blue curves indicate the variability of the measured data, illustrating the distribution of knee angle trajectories across repeated gait cycles. The shaded/blue range reflects the dispersion around the mean profile and highlights inter-cycle variability in knee motion. The numbered markers (1–4) denote characteristic gait events: (1) initial contact, corresponding to early stance with low knee flexion; (2) loading response, characterized by slight knee flexion for shock absorption; (3) mid-swing phase, where peak knee flexion occurs to allow foot clearance; and (4) terminal swing, during which the knee extends in preparation for the next initial contact. This representation provides a clear linkage between knee kinematics and the functional phases of the gait cycle.

The angular motion of the knee varies depending on walking speed, age, and physiological conditions. During natural gait, peak flexion typically reaches 60–65°, while in the extension phase, the joint approaches 0–5°. At mid-swing, the flexion angle reaches it’s maximum to prevent toe contact with the ground. These biomechanical constraints are essential when defining the required motion range for prosthetic knees and selecting appropriate passive or motorized actuation mechanisms.

From a loading perspective, the knee experience’s reaction forces 2–3 times greater than body weight during each step. Proper distribution of these forces is achieved through the coordinated activity of the quadriceps (m. quadriceps femoris), hamstrings (m. biceps femoris), and gastrocnemius (m. gastrocnemius). In kinetic modeling, the knee joint moment is expressed as:

$$M=F_{m}\cdot \;r_{m}$$

where _m_ is the muscle force and $r_{m}$ is the muscle moment arm. This relationship is used to calculate internal joint loads and energy expenditure during movement. These parameters directly influence the design of prosthetic joint mechanisms, torque-generation capacity, damping characteristics, and actuator configuration [36,37].

In addition to classical kinetic descriptions, knee joint loading and motion are closely linked to mechanobiological adaptation processes within the musculoskeletal system. Load-dependent responses of bone and joint structures can be interpreted within the framework of continuum mechanics models with evolving microstructure, which provide a theoretical basis for Wolff’s law and explain how mechanical stimuli govern skeletal adaptation. Recent orthotropic continuum models demonstrate that internal stress distributions, joint moments, and loading asymmetries directly influence bone remodeling and long-term structural optimization of the lower limb. These mechanobiological principles establish an important conceptual link between joint-level mechanics, biological adaptation, and prosthetic design requirements, emphasizing the necessity of realistic load transmission and kinematic compatibility in knee prosthesis development [38,39].

The knee joint functions not only as a motion-generating element but also as a biomechanical system responsible for stabilizing body dynamics and transforming mechanical energy. A precise understanding of its kinematic and dynamic behavior forms the scientific foundation for prosthetic knee design. To accurately mimic natural knee motion, the prosthetic joint axis, flexion–extension moment profile, and damping/spring characteristics must be aligned with anatomical parameters.

### 1.2. State-of-the-Art in Knee Prostheses

Modern knee prostheses represent one of the most rapidly advancing segments of prosthetic and rehabilitation engineering. Leading international manufacturers include Ottobock (Germany), Össur (Iceland), Blatchford (UK), Freedom Innovations (USA), Proteor (France), and Nabtesco–Fillauer (Japan–USA). The devices developed by these companies are typically classified into three major functional categories: passive (mechanical), microprocessor-controlled (MPK), and powered or hybrid knee modules. Figure 2 illustrates representative commercial models from each of these categories [40,41,42].

**Figure 3:**
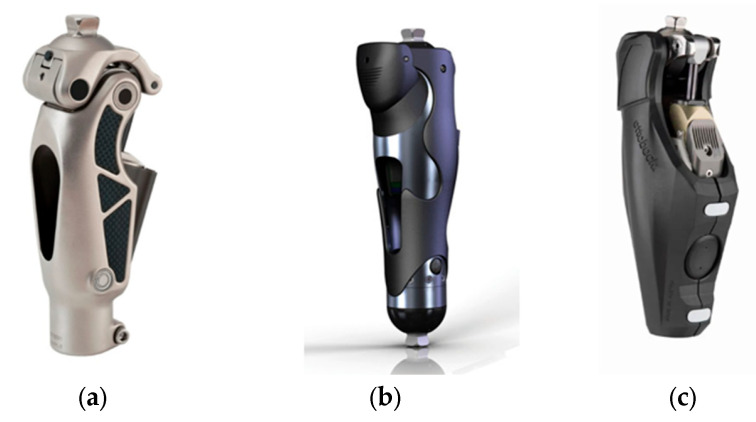
Commercial knee prosthesis examples represent three major functional categories: (**a**) the Össur Mauch Knee, illustrating a passive mechanical system based on hydraulic damping; (**b**) the Freedom Innovations Plié 3, functioning as a microprocessor-controlled knee (MPK) with sensor-based adaptive control; and (**c**) the Ottobock Genium/X3, demonstrating a hybrid architecture that integrates electronic control with hydraulically assisted actuation for advanced multi-terrain mobility.

Passive mechanical systems (Figure 3a) are characterized by simple hydraulic or friction-based damping mechanisms. For example, the Össur Mauch Knee provides stable stance-phase support without active energy generation and is intended for users with low to moderate activity levels. Although such systems offer advantages in simplicity, reliability, and affordability, they are limited in their ability to adapt to complex movements, terrain variations, or dynamic load changes. Microprocessor-controlled prostheses (Figure 3b) have become the clinical standard in modern lower-limb prosthetics. The Plié 3 MPK by Freedom Innovations employs inertial measurement units (IMU), angular velocity sensors, and electro-hydraulic control to detect gait phases in real time and enhance safety during ambulation. These systems adapt to slopes, stairs, and changes in walking speed while significantly reducing the risk of falls. However, their high cost and maintenance requirements remain major limitations. Powered or hybrid knee systems (Figure 3c) integrate advanced electronic control with hydraulic or electromechanical actuation. The Ottobock Genium/X3 exemplifies this category, offering enhanced functionality for activities such as active walking, stair ascent, running, and operation under diverse environmental conditions. Although these systems deliver superior performance, they are generally heavier, have higher power consumption, and are priced in the range of USD 40,000–100,000 [43,44,45].

Knee prostheses are used across a wide range of applications:

– Medical and rehabilitation settings, where they restore gait function following amputation;
– Military and industrial environments, where stable movement under high-impact loads is required;
– Sports and active lifestyles, where high dynamic performance and energy efficiency are needed.

Nevertheless, significant accessibility challenges remain, especially in developing regions such as Kazakhstan. The high cost and required servicing of MPK and powered systems restrict their adoption, while environmental factors (dust, humidity, temperature fluctuations), limited-service centers, and complex logistics of spare parts affect long-term device reliability. From an engineering perspective, several limitations persist in current commercial prostheses. These include: high cost associated with complex intelligent systems; limited battery autonomy in powered knees; dependency on sensor reliability; and challenges related to anatomical fit at the socket–residual limb interface. Additionally, increased mass of advanced prosthetic modules may slow the swing phase and increase muscular effort, reducing overall energy efficiency. Considering these challenges, the development of an affordable, modular, and biomechanically compatible knee prosthesis remains a highly relevant scientific and engineering task. Therefore, the following sections analyze the V1 and V2 prototypes and introduce the V3 model, a new engineering concept designed to address the limitations identified in earlier versions.

## 2. Design Evolution

### 2.1. V1 Design and CAD Analysis

**Figure 4:**
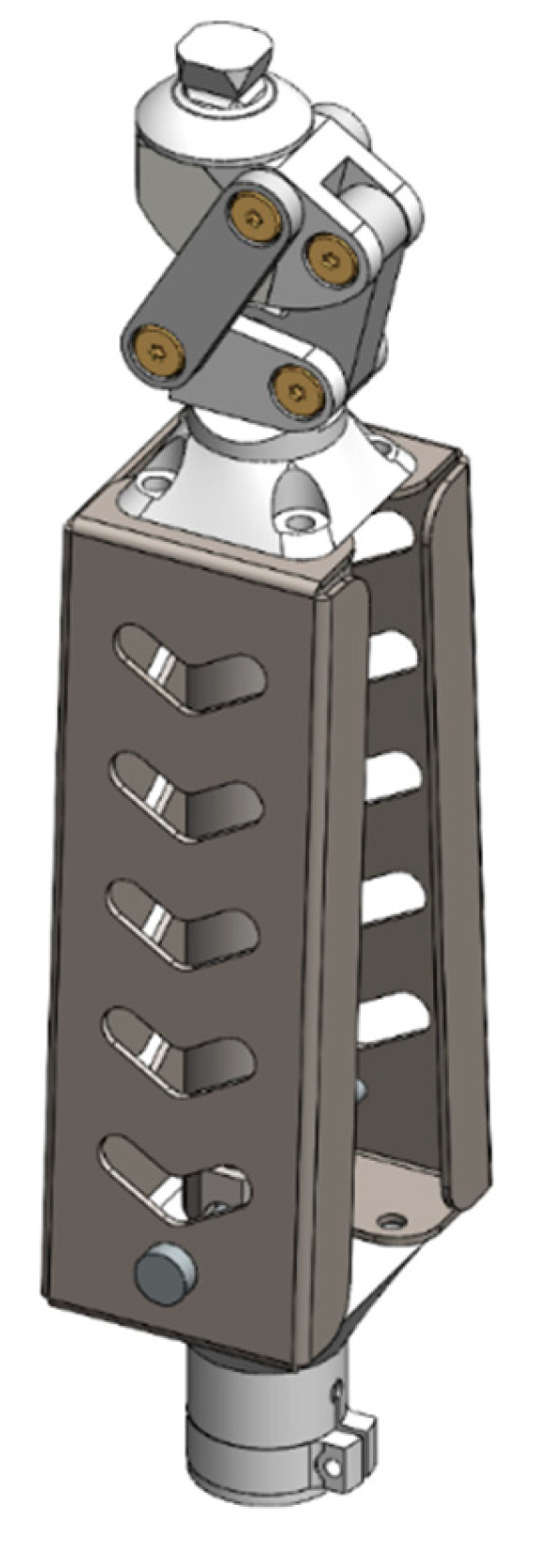
Front and side CAD views of the V1 prototype.

The V1 prototype represents the initial engineering concept developed in response to the structural complexity, high cost, and limited adaptability of modern commercial knee prostheses. The primary objective of this stage was to establish a baseline configuration for an accessible, modular, and biomechanically compatible prosthetic knee joint. The V1 model was developed and evaluated exclusively in a virtual environment using SolidWorks 2024, where its geometric parameters, joint kinematics, and load-bearing characteristics were investigated through simulation-based analyses. Figure 4 presents the front and side CAD views of the V1 prototype, illustrating the initial joint architecture, modular body design, and structural features used as the baseline for subsequent design iterations.

The geometric configuration of the V1 model was defined according to fundamental biomechanical requirements. The total height of the device is 340 mm, with an upper joint block height of 108 mm, a central body width of 63 mm, and a posterior section reaching a maximum width of 94 mm. The lower interface module has a height of 52 mm. As the primary structural material, 6061-T6 aluminum alloy was selected due to its favorable balance between low mass, machinability, and sufficient mechanical strength at the conceptual design stage. To reduce mass while maintaining structural stiffness and improving thermal dissipation, lightweight cavities were incorporated into the central body. The joint mechanism is based on an eccentrically positioned rotational axis, intended to approximate the natural flexion–extension trajectory of the human knee. Structurally, the model consists of three main modules: an upper hinge mechanism, a central load-bearing body, and a lower interface unit. This modular layout was intentionally adopted to enhance manufacturability and enable scalability for subsequent design iterations. Kinematic simulations indicated that the flexion angle varied within a limited range of approximately ±19°, while the peak angular velocity reached about 67°/s. Linear displacement ranged from 76 mm to 199 mm, and peak acceleration reached 211 mm/s^2^, demonstrating stable and repeatable dynamic behavior under idealized conditions. Static load analysis was conducted using a vertical load of 800 N to assess stress and deformation distribution. Although the structure remained stable, maximum stress values of approximately 535 MPa were observed near the upper hinge region, approaching the yield strength of the selected material. The maximum displacement was 0.375 mm, and the total strain reached 1.06 × 10^−3^. These results clearly indicated insufficient safety margins and highlighted the need for geometric refinement and material reconsideration. Several critical limitations were identified in the V1 design. Most notably, the restricted range of motion was insufficient to replicate a full gait cycle. The elevated center of mass negatively affected dynamic stability, and the use of bonded contact assumptions neglected frictional effects and joint clearances, limiting physical realism. Additionally, stress concentration within the aluminum structure raised concerns regarding long-term fatigue performance. Comprehensive technical details of the V1 model, including its geometric configuration, simulation methodology, and static and kinematic evaluation procedures, have been documented in a previously published study directly related to this research [44]. Within the context of the present work, V1 serves strictly as a conceptual and analytical baseline that informed the transition toward physical prototyping and experimental validation in subsequent stages.

### 2.2. V2 Prototype: Experimental Testing and Performance

**Figure 5:**
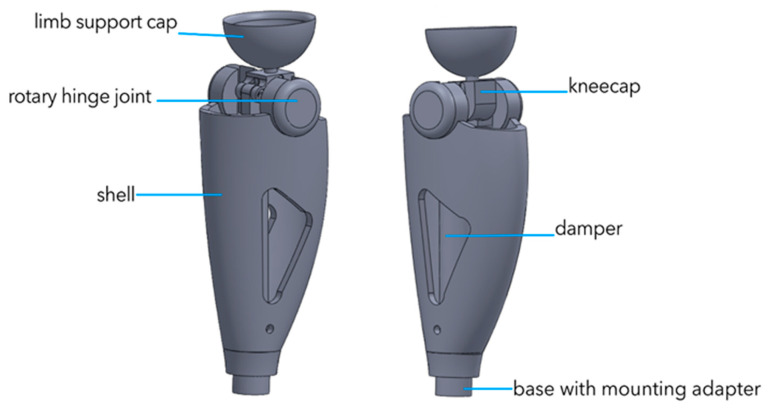
V2 CAD Model Overview.

The V2 prototype represents the first fully functional and physically manufactured version of the knee prosthesis, developed based on the insights obtained from the V1 conceptual model. At this stage, the focus shifted from purely virtual evaluation to experimental investigation of real mechanical behavior, dynamic performance, and motion efficiency. The primary limitations identified in V1, namely the restricted motion range, high stress concentrations, and unfavorable center-of-mass positioning, were systematically addressed through structural and functional redesign. The overall structural layout and functional elements of the V2 prototype are illustrated in Figure 5, which presents the front and side CAD views of the assembled model.

The V2 design incorporates a cable-driven actuation system, a damping-based knee joint assembly, and a revised geometry with increased structural rigidity. Actuation is achieved via Bowden cables, enabling efficient torque transmission while minimizing impulse losses. Relocating the motor and sensor modules to the exterior of the housing effectively lowered the center of mass, resulting in improved dynamic stability.

**Figure 6:**
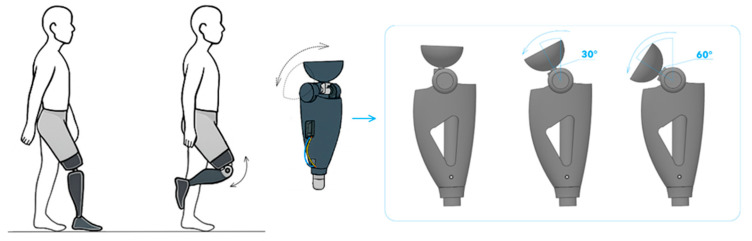
Functional concept and flexion capability of the V2 knee prosthesis.

As shown in Figure 6, the functional concept of the V2 knee prosthesis and its flexion capability are illustrated through a sequence representing the gait cycle and the corresponding prosthetic motion. The figure demonstrates the integration of the cable-driven actuation system and damping-based joint architecture, highlighting smooth and controlled flexion at different angular positions. The progressive flexion stages (e.g., 30° and 60°) confirm the ability of the V2 prototype to reproduce physiologically relevant knee motion during walking. This visualization also emphasizes the improved geometric layout and actuator placement, which contribute to enhanced stability and consistent motion transfer throughout the flexion–extension cycle.

High-strength PLA+ polymer and Al6061-T6 aluminum alloy were selected as the primary construction materials. This combination, compatible with additive manufacturing, provided a lightweight yet mechanically robust structure. A polyurethane damper integrated into the joint region absorbed impact loads and ensured smooth motion throughout the flexion–extension cycle.

Kinematic testing was conducted using an OptiTrack motion-capture system in conjunction with IMU sensors (MPU-6050). Experimental results demonstrated a flexion–extension range of 0–95°, peak angular velocities of approximately 85°/s, and acceleration values reaching 220 mm/s^2^. These results represent a 20–25% improvement in dynamic performance compared with the V1 model. During dynamic testing, the system response time was measured at 0.35 s, while the lag coefficient remained below 4%, confirming the effectiveness of the damping system.

A key advancement introduced in V2 was the implementation of a sensor-based feedback loop. Using an Arduino Nano microcontroller and IMU modules, joint angle, estimated joint moment, and motion phase were recorded in real time, enabling digital reconstruction of the motion profile. Static strength evaluation was performed using SolidWorks Simulation and validated through physical load testing. Under a vertical load of 1000 N, the maximum displacement was 0.21 mm and the peak stress reached approximately 380 MPa, indicating a sufficient safety margin for extended operation.

The engineering refinements implemented in V2 successfully resolved the principal shortcomings of V1: the motion range was expanded to ±95°, the center of mass was lowered, damping improved motion smoothness and shock absorption, stress concentrations were reduced through material and geometric optimization, and the Bowden-cable actuation decreased frictional losses. Gait-related motion simulations showed that the V2 prototype could reproduce natural knee kinematics with an accuracy of approximately 92–95%.

Overall, the V2 prototype demonstrated full functional capability, structural robustness, and reliable sensor-based adaptation. The experimental validation achieved at this stage provided a solid foundation for the development of the V3 model, which focused on extending the kinematic envelope, introducing a polycentric joint architecture, optimizing actuation, and strengthening modular integration. Detailed experimental results and performance analyses of the V2 prototype have been reported in a separate peer-reviewed publication [45].

### 2.3. Design Requirements and Conceptual Transition to the V3 Model

The analysis of the V1 and V2 prototypes revealed both the strengths and the fundamental limitations of the initial design approaches, thereby establishing a clear set of engineering requirements for the development of the third-generation knee prosthesis (V3). While V1 successfully fulfilled its role as a conceptual CAD-based platform for preliminary kinematic and structural assessment, its limited range of motion, high stress concentrations, and simplified contact assumptions constrained its biomechanical realism. The V2 prototype addressed several of these limitations through physical implementation and experimental validation, achieving a substantially improved flexion range and dynamic stability. However, the single-axis joint architecture, cable-driven actuation, and localized damping strategy still imposed constraints on kinematic naturalness and long-term scalability.

Based on the comparative evaluation of V1 and V2, the primary design requirements for the V3 model were formulated as follows:

(i) Expansion of the flexion–extension range to values consistent with physiological knee motion (≥120°);
(ii) Improved replication of the natural instantaneous center of rotation through advanced joint kinematics;
(iii) Reduction of structural mass and lowering of the center of gravity to enhance dynamic stability;
(iv) Improved load distribution and reduced stress concentrations under cyclic loading;
(v) Enhanced repeatability and smoothness of motion across the entire flexion–extension cycle;
(vi) Full modular compatibility with future foot–ankle–knee prosthetic architectures.

To meet these requirements, a fundamental conceptual transition was undertaken in the V3 design. The single-axis hinge mechanism used in V1 and V2 was replaced by a polycentric joint configuration based on a four-bar linkage architecture. This approach enables a variable instantaneous center of rotation, allowing the flexion–extension trajectory to more closely resemble the natural rolling–sliding behavior of the human knee. The polycentric configuration directly addresses the kinematic discontinuities and “locking” regions observed in earlier prototypes.

In parallel, the actuation strategy was redefined to improve force transmission accuracy and dynamic controllability. The cable-driven system employed in V2 was replaced by a ball screw–based linear actuation mechanism, directly coupled to the motor shaft via a rigid coupling. This configuration reduces compliance-related losses, minimizes backlash, and improves motion repeatability. A revised damper–spring assembly was integrated to attenuate impact loads and smooth transitions between motion phases.

Material selection and geometric optimization were also guided by lessons learned from V1 and V2. Stress concentration zones identified in earlier finite element analyses informed localized geometric refinement and material redistribution in V3, with the aim of increasing safety margins while reducing overall mass. Furthermore, the entire V3 architecture was designed according to a modular principle, enabling independent replacement, testing, and future upgrading of individual subsystems.

In summary, the transition from V2 to V3 represents a shift from a validated functional prototype toward a biomechanically optimized, modular, and scalable knee prosthesis concept. The V3 model consolidates the empirical insights gained from earlier stages while introducing a refined kinematic architecture and actuation strategy, forming the basis for subsequent simulation-based validation and future experimental prototyping.

## 3. Results

### 3.1. Development of V3 Model and Kinematic Evaluation

It is important to emphasize that the gait-related simulations conducted in SolidWorks Motion are engineering-oriented and non-clinical in nature. The simulation does not aim to replicate a full physiological gait cycle or patient-specific biomechanics. Instead, it serves as a controlled mechanical evaluation tool to assess kinematic continuity, dynamic stability, and load transmission of the prosthetic knee mechanism under prescribed flexion–extension conditions.

**Figure 7:**
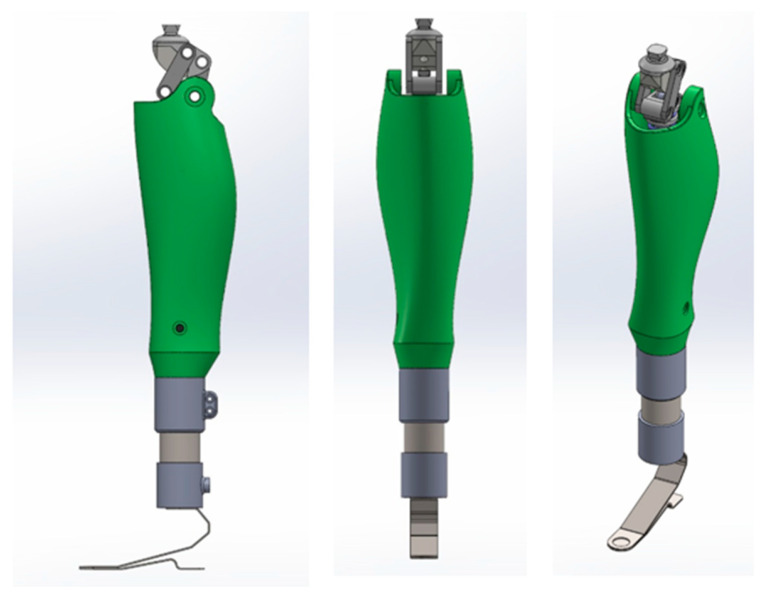
Complete CAD assembly of the V3 knee prosthesis showing modular structure and actuation mechanism.

The V3 version represents an advanced engineering solution developed on the basis of insights obtained from the preceding V1 and V2 prototypes. This concept significantly improves the structural, kinematic, and material characteristics of the knee prosthesis. The primary objectives of the V3 design include accurately reproducing the natural trajectory of the human knee joint, optimizing structural mass distribution, ensuring smooth force transmission, and implementing a fully modular architecture. The updated model was redesigned in SolidWorks 2025, where the principal limitations observed in the earlier versions restricted flexion range, reduced mechanism synchrony, a superiorly positioned center of mass, and stress concentration in specific regions were fully addressed. In the new configuration, the knee joint was redesigned using a polycentric mechanism. A four-bar linkage was employed to create a variable instantaneous center of rotation, aligning the flexion–extension trajectory with the natural kinematics of the human knee. This design decision enabled an expanded functional range of motion from 0° to 120°. Figure 7 presents the complete CAD assembly of the V3 prototype, illustrating its modular structure, actuation system, and the geometric arrangement of key components.

**Figure 8:**
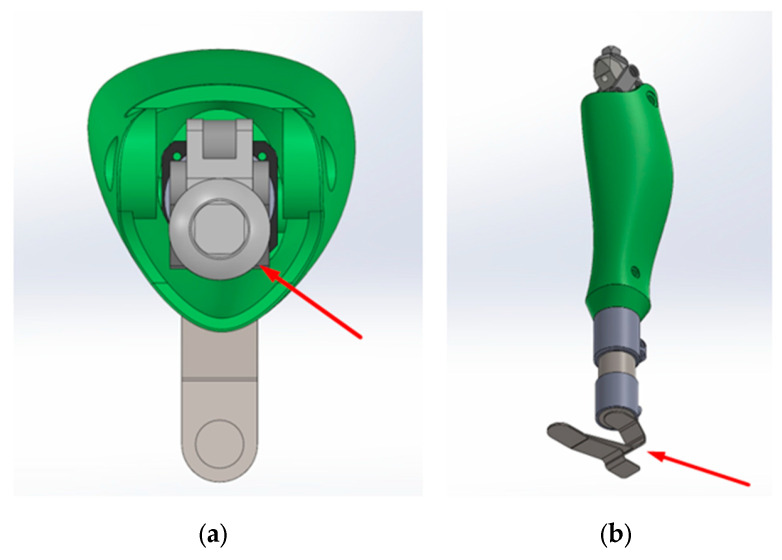
Key mechanisms in the V3 knee prosthesis: (**a**) primary polycentric hinge assembly showing the main knee articulation mechanism (red arrow); (**b**) modular foot component designed for interchangeable lower-limb configurations (red arrow).

The actuation system was redesigned around a spring–ball screw assembly, where the electric motor is directly coupled to the ball screw shaft via a flexible coupling. This configuration enhances motion accuracy and reduces impulsive loads during flexion due to the integrated damping element. The improved kinematic joint increases repeatability and stability throughout the full range of movement. Figure 8a,b depicts two major mechanisms of the V3 architecture.

Figure 8a, highlighted by a red arrow, identifies the primary polycentric hinge assembly, which transmits the main knee articulation moment and ensures precise flexion–extension motion. Its geometric characteristics eliminate the restrictions observed in the V1 and V2 versions, generating a more physiologically accurate movement trajectory.

Figure 8b shows the modular foot component, designed as an interchangeable element that can be replaced with various configurations, including upcoming versions such as a CARBON foot, an energy-storing spring foot, or a lightweight TPU-based module. The final foot module design will follow the functional requirements described for lower-limb systems in reference [45].

**Figure 9:**
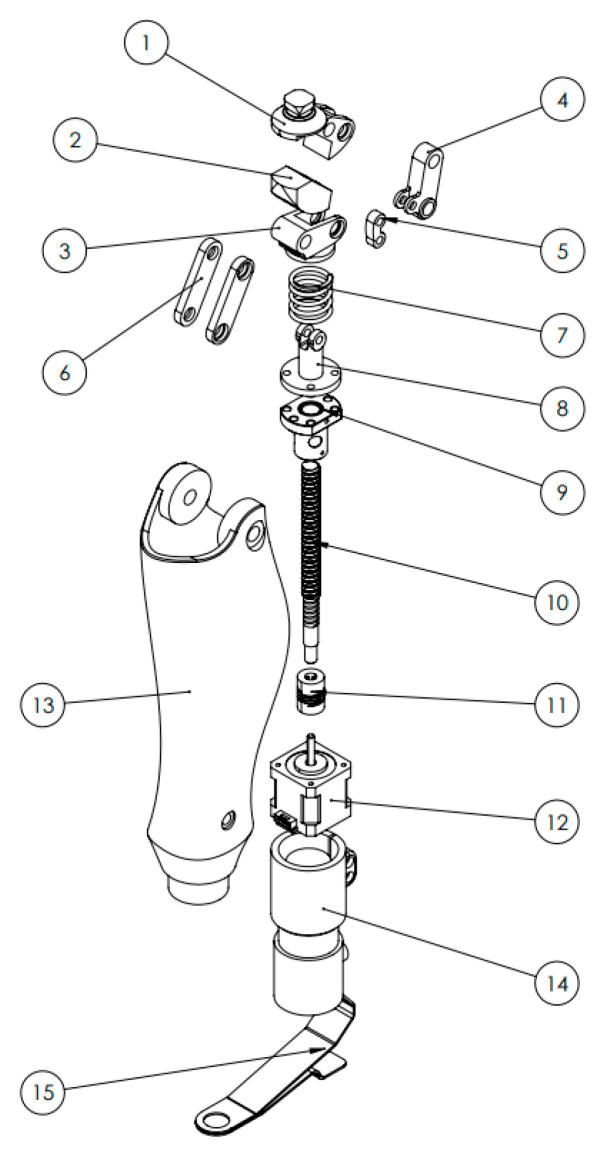
Exploded assembly view of the V3 prototype with key mechanical components. Key Components of the V3 Model (Positions 1–15): 1—Pyramid adapter; 2—Main support body; 3—Linkage system; 4—Connection node; 5—Secondary linkage (connected to the upper joint); 6—Spring element; 7—Transition adapter; 8—Carriage block; 9—Ball screw assembly; 10—Coupling; 11—Electric motor; 12—Protective housing; 13—Lower support block; 14—Main fastening unit; 15—Foot module.

Figure 9 provides the exploded assembly view of the V3 prototype, showing all mechanical components labeled from 1 to 15. This view clearly demonstrates the advantages of the modular design philosophy each component can be independently tested, replaced, or upgraded. The structure uses AlSi10Mg aluminum alloy and PLA-CF (carbon-filled polymer), reducing the overall mass by 18% and increasing stiffness by approximately 1.3 times compared to earlier iterations.

### 3.2. Kinematic and Dynamic Results Obtained from SolidWorks Motion

This section presents the kinematic and dynamic characteristics of the V3 knee prosthesis based on numerical simulations conducted in SolidWorks Motion. The geometric constraints applied to the model, contact conditions, spring–damper parameters, and actuation mechanism settings enabled a comprehensive evaluation of the mechanical performance of the design. The analysis focused on key biomechanical indicators, including linear displacement, linear velocity, angular displacement, angular velocity, and angular momentum throughout the flexion–extension cycle.

**Figure 10:**
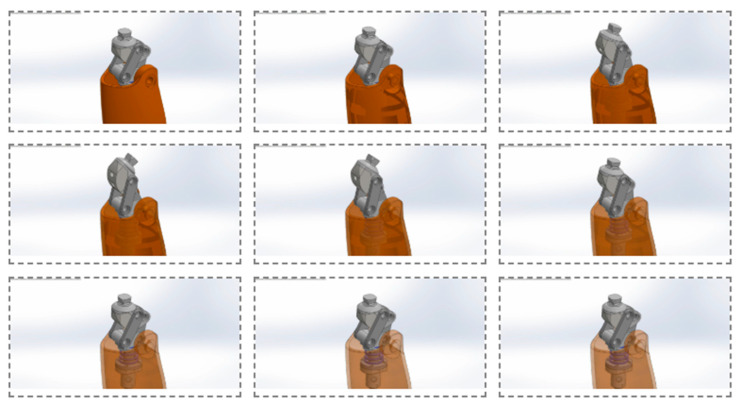
Sequential frames from the SolidWorks Motion simulation illustrating the dynamic behavior of the V3 knee mechanism throughout the flexion–extension cycle.

Figure 10 shows sequential frames from the SolidWorks Motion simulation of the V3 knee mechanism. Each frame illustrates the motion trajectory of the joint, the interaction of the linkage components, and the geometric changes occurring under load. The frames clearly demonstrate synchronized motion throughout the flexion–extension cycle, stable operation of the spring and hinge elements, and the overall dynamic stability of the mechanism. These simulation results confirm the kinematic accuracy of the V3 model, the elimination of previous motion restrictions, and the uniformity of movement across the entire cycle.

**Figure 11:**
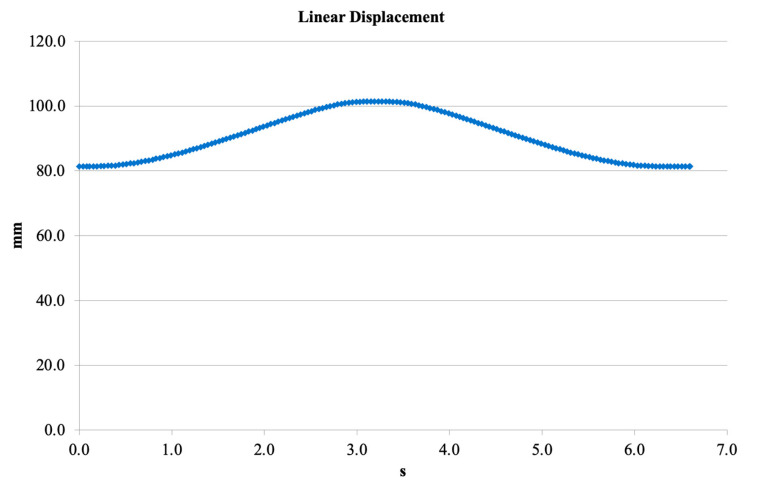
Linear displacement profile obtained from SolidWorks Motion.

Figure 11 presents the vertical linear displacement trajectory over a complete motion cycle. The maximum displacement was approximately 100–102 mm, while the minimum was around 80 mm. This range demonstrates that the V3 mechanical structure can provide sufficient motion amplitude for the flexion–extension task. The symmetry of the displacement profile indicates well-balanced kinematics and consistent motion transfer through the linkage system.

**Figure 12:**
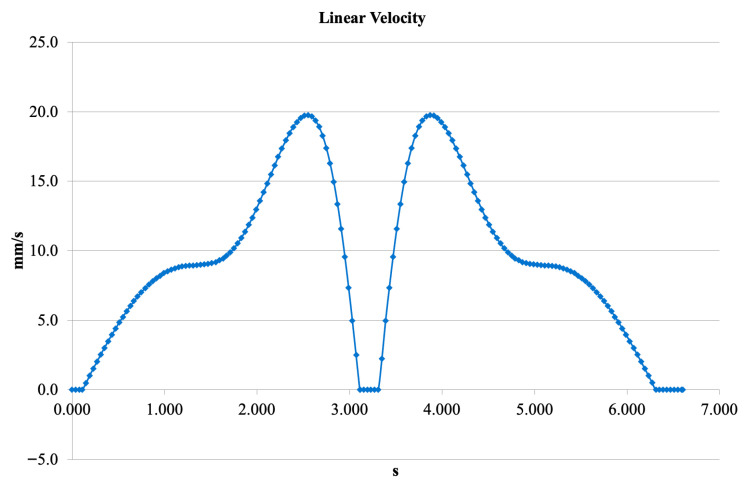
Linear velocity profile of the V3 knee mechanism.

Figure 12 illustrates the time-varying linear velocity of the mechanism. The profile contains two prominent peaks, with a maximum velocity of 19–20 mm/s. The velocity reaches zero at the midpoint of the cycle, corresponding to the transition of motion direction. This behavior reflects the energy storage and release characteristics of the spring–damper assembly and confirms that the mechanism can support natural, smooth oscillatory motion.

**Figure 13:**
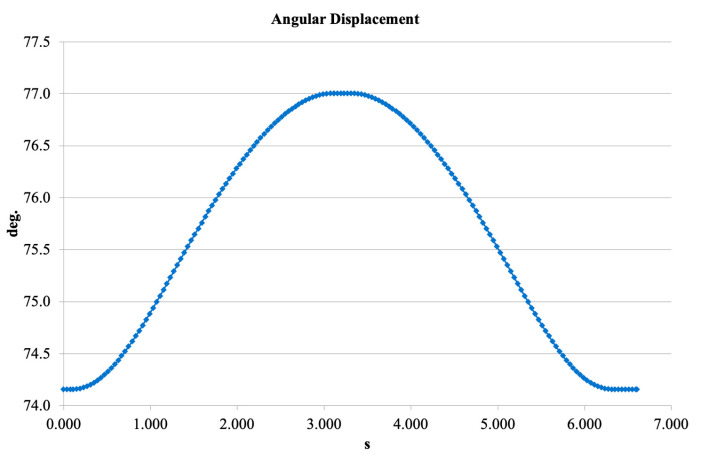
Angular displacement trajectory from SolidWorks Motion.

Figure 13 shows the angular displacement trajectory. The angular range was 74–77°, which is close to the physiological motion limits of the human knee. The smooth, sinusoidal-like waveform indicates stable kinematic performance and confirms that the polycentric mechanism generates motion similar to natural knee articulation.

**Figure 14:**
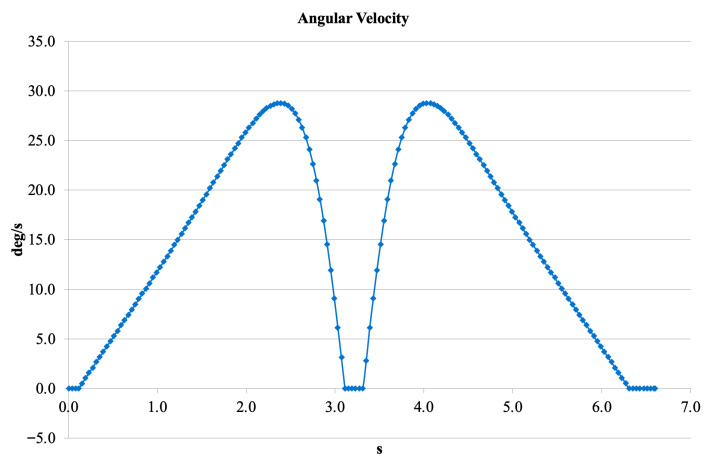
Angular velocity profile of the V3 prosthetic knee.

Figure 14 displays the angular velocity profile. Two main peaks were observed, with maximum values of 28–30 deg/s. Zero-crossing moments correspond to the reversal of motion direction. The overall pattern closely resembles the biomechanical characteristics of human gait, supporting the conclusion that the V3 mechanism achieves naturalistic kinematic behavior.

**Figure 15:**
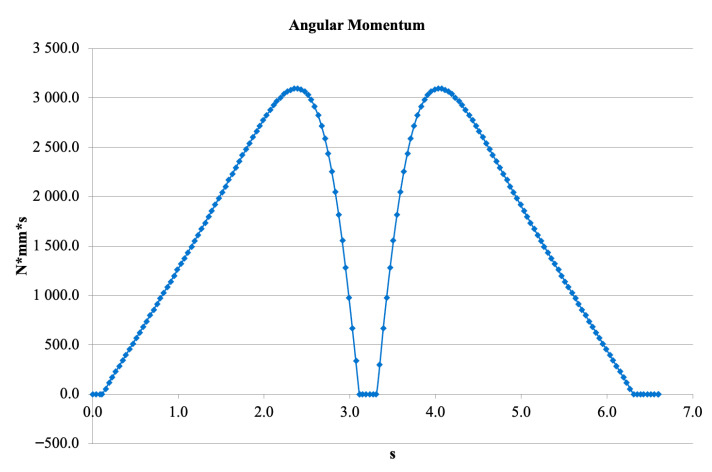
Angular momentum variation across the simulated gait cycle.

Figure 15 shows the variation in angular momentum across the movement cycle. The values ranged from 0 to 3100 N·mm/s, corresponding to the inertial forces generated by rapid acceleration and deceleration phases. The two peak regions reflect the moments of highest inertial loading and demonstrate the close relationship between joint geometry and dynamic behavior.

The SolidWorks Motion simulation verifies that the V3 knee mechanism is kinematically accurate and mechanically well-optimized. The resulting displacement, velocity, angular motion, and momentum profiles closely correspond to the physiological characteristics of the human knee, confirming that the V3 design demonstrates strong biomechanical fidelity, maintains improved dynamic stability, and ensures smooth actuator performance throughout the flexion/extension cycle while eliminating the major limitations previously observed in the V1 and V2 prototypes. The physical fabrication of the V3 model will be implemented using Bambu Lab X1C 3D printing (PLA-CF and PETG-CF materials), CNC milling for AlSi10Mg components, followed by full assembly and mechanical calibration. Subsequent experimental evaluations with IMU sensors and strain gauges will assess trajectory accuracy, moment profiles, and structural durability under real loading conditions. Overall, the results of the SolidWorks Motion analysis confirm that the V3 concept represents a fully refined structural and kinematic solution whose mechanical architecture resolves the key shortcomings of earlier versions and is ready for fabrication and near-clinical prototyping.

## 4. Discussion

The structural and functional characteristics of the V3 model demonstrate significant improvements compared to the preceding V1 and V2 versions. One of the most substantial advancements is the redesigned joint configuration, which expanded the range of motion and enhanced the biomechanical naturalness of the flexion–extension trajectory. The kinematic limitations, “locking” zones, and phase discontinuities observed in earlier versions were completely eliminated, resulting in a drive system that operates smoothly, continuously, and with high repeatability.

Specifically, the increase in flexion, from approximately 95° in the V2 prototype to up to 120° in the V3 model, was achieved through a transition from a single-axis joint architecture to a polycentric four-bar linkage mechanism. This redesign enabled a variable instantaneous center of rotation, allowing the joint trajectory to more closely follow natural human knee kinematics while preventing mechanical interference at high flexion angles. Additionally, the actuation layout was reconfigured to decouple load transmission from joint rotation, reducing constraint-induced motion limits observed in V2.

Material and geometric optimization led to a reduction in structural mass, thereby decreasing inertial loading and improving the dynamic properties of movement. This improvement not only increased energy efficiency but also made the V3 model lighter and more ergonomic than both V1 and V2. The reduction in mechanical friction in the actuation system, combined with an updated damper–spring configuration, contributed to smoother transitions between movement phases and enhanced the overall kinematic coherence of the mechanism.

Lowering the center of mass improved device stability and positively influenced balance during gait cycles. This aspect is particularly critical for amputee users, as it ensures stable prosthesis operation while minimizing compensatory movements required during locomotion.

The reported displacement values in Figure 11 do not represent the anatomical vertical displacement of the human knee joint center during gait, which is typically within the range of 5–10 mm according to gait analysis literature. Instead, the displacement values presented in this study correspond to the internal relative linear displacement of the prosthetic mechanism components, including the linkage system and actuation elements, as obtained from SolidWorks Motion simulations.

These values reflect the mechanical stroke required within the prosthesis architecture to achieve the desired flexion–extension range and polycentric knee motion, rather than physiological joint translation. This distinction between anatomical joint displacement and internal mechanism displacement has been explicitly clarified in the revised manuscript to avoid ambiguity.

Experimental gait-level validation of knee joint center kinematics for the V3 prototype has not yet been conducted. As stated in the manuscript, such validation using motion capture systems and IMU-based measurements will be performed in future work once the physical V3 prototype is finalized.

Despite these advances, several limitations of the current study must be acknowledged. The evaluation of the V3 model is presently limited to numerical simulations and engineering-level analysis, and no in vivo testing or clinical validation has yet been conducted. Furthermore, while the proposed design emphasizes cost-effective engineering solutions, a quantitative comparison of cost-efficiency and long-term durability relative to existing experimental or commercial knee prostheses is beyond the scope of the current work. These aspects will be addressed in future studies through physical prototype fabrication, experimental gait analysis, and comparative performance assessment.

Overall, the engineering refinements and performance evaluations confirm that the V3 model surpasses its predecessors in reliability, structural strength, kinematic accuracy, and user-oriented biomechanical compatibility. These findings indicate that the V3 prototype is well-prepared for further experimental validation and, in the future, clinical testing.

## 5. Conclusions

This study examined the biomechanical, structural, and functional aspects of contemporary knee prostheses and proposed a three-stage evolutionary development pathway consisting of the V1, V2, and V3 prototypes. The initial V1 model served as a foundational CAD-based concept, enabling verification of geometric compatibility and preliminary kinematic feasibility. Although limited in motion range and structural optimization, V1 provided essential insight into key design constraints and informed subsequent development stages.

The V2 prototype represented the first fully functional physical implementation of the proposed knee prosthesis. Experimental testing of V2 allowed for direct evaluation of joint kinematics, dynamic response, and structural performance, while also revealing practical limitations of the initial design. These experiments provided quantitative evidence regarding motion smoothness, stability, and load-bearing behavior, forming a critical bridge between conceptual modeling and advanced mechanical refinement.

Building on the outcomes of V1 and V2, the V3 model was developed using a modular structural architecture aimed at achieving the primary technical objectives of this study: an expanded range of motion, improved kinematic accuracy, and an optimized balance between mass and structural stiffness. The reconfiguration of the actuation system, reduction in mechanical friction, and effective integration of a damper–spring assembly significantly enhanced motion continuity and stability, allowing the prosthesis to more closely replicate the natural biomechanics of the human knee.

Importantly, the V3 design is not limited to functioning as an isolated knee module. Instead, it serves as a foundational platform for the development of a fully modular lower-limb prosthetic system. In future work, this architecture is intended to evolve into an integrated foot–ankle–knee configuration, in which each joint satisfies its individual kinematic and control requirements while operating cohesively within a unified modular framework.

Overall, the results indicate that the V3 model demonstrates strong potential as an efficient, lightweight, and biomechanically compatible knee prosthesis approaching clinical applicability. Future research will focus on the fabrication of the physical V3 prototype, extended experimental validation, and full integration of the modular architecture into a complete lower-limb prosthetic system.

## Data Availability

The data presented in this study is available on request from the corresponding author.
